# Outcomes of cases with elevated 3-hydroxyisovaleryl carnitine report from the newborn screening program

**DOI:** 10.1016/j.ymgmr.2024.101153

**Published:** 2024-10-13

**Authors:** Fuad Al Mutairi, Randa Alkhalaf, Abdul Rafiq Khan, Ali Al Othaim, Majid Alfadhel

**Affiliations:** a Genetic and Precision Medicine Department, King Abdullah Specialized Children Hospital, King Abdulaziz Medical City, Ministry of National Guard Health Affairs (MNGHA), Riyadh, Saudi Arabia; b Department of Pathology and Laboratory Medicine, King Abdulaziz Medical City, Riyadh, Saudi Arabia; c King Abdullah International Medical Research Center (KAIMRC), King Saud bin Abdulaziz University for Health Sciences, Ministry of National Guard Health Affairs, Saudi Arabia

**Keywords:** Newborn screening, C5OH, 3-methylcrotonyl-CoA carboxylase deficiency, β-Ketothiolase deficiency, HMG-CoA lyase deficiency

## Abstract

**Background:**

Elevated plasma levels of 3-hydroxyisovaleryl-carnitine (C5OH) and impaired leucine catabolism are frequently observed in newborn screening reports, necessitating consideration of various diseases in the differential diagnosis. This study aimed to analyze different forms of C5OH and explore their potential predictive value for diagnosis and outcomes.

**Methods:**

A retrospective review of newborn screening positive cases for C5OH-related diseases from May 2011 to December 2023 was conducted. Clinical, biochemical, and molecular phenotypes of all confirmed positive cases during this period were examined.

**Results:**

A total of 15 true positive cases were diagnosed. No significant correlation was found between the C5OH levels in newborn screening and the diagnosis of specific C5OH-related disorders or the presence of metabolic, neonatal, or developmental abnormalities. Outcomes varied based on the spectrum of diseases.

**Conclusion:**

These findings indicate that relying solely on C5OH levels from newborn screening is insufficient for making accurate diagnoses or predictions regarding C5OH-related disorders. Further comprehensive evaluation and consideration of additional factors are essential for accurate diagnosis, management and outcome.

## 1 Background

The newborn screening (NBS) program has been instrumental in the early detection of inborn errors of the metabolism (IEM), but there is no agreement on the components of neonatal metabolic screening panels [1]. In the 1990s, an expanded NBS using tandem mass spectrometry (MS/MS) was implemented to detect treatable IEM shortly after birth. This method measures acylcarnitine profiles and amino acids, expanding the range of metabolic diseases tested from asymptomatic individuals to those with severe symptoms [2,3]. In Europe, experts are evaluating the NBS program to identify and recommend a core group of diseases for screening [4]. However, differences in conditions and factors such as epidemiological data, cultural perceptions, and economic considerations have led to various recommendations across countries [5]. For instance, while many Middle Eastern and North African countries have high coverage for congenital hypothyroidism, there is no consensus on the diseases to be included in NBS by MS/MS [6].

3-Hydroxyisovalerylcarnitine (C5OH) is a significant acylcarnitine parameter in NBS tests. Its elevation is a strong indicator of organic acidemias in the leucine pathway, such as 3-methylcrotonyl-CoA carboxylase (3MCC) deficiency, 3-hydroxymethyl-3-methylglutaryl-coenzyme A lyase (HMG-CoA lyase) deficiency, β-ketothiolase deficiency, and multiple carboxylase deficiency (MCD). In biotinidase deficiency, elevated levels of C5-OH acylcarnitine are typically observed when the patient is a few months old, in contrast, holocarboxylase synthetase deficiency may lead to an early increase in C5-OH acylcarnitine levels, often within the first few days or weeks of life [7,8]. The Centers for Disease Control and Prevention's (CDC) Newborn Screening Quality Assurance Program includes C5OH testing in its dried blood spot (DBS) proficiency testing and quality control materials, promoting standardization of C5OH measurements across newborn screening laboratories worldwide [9].

**Fig. 1:**
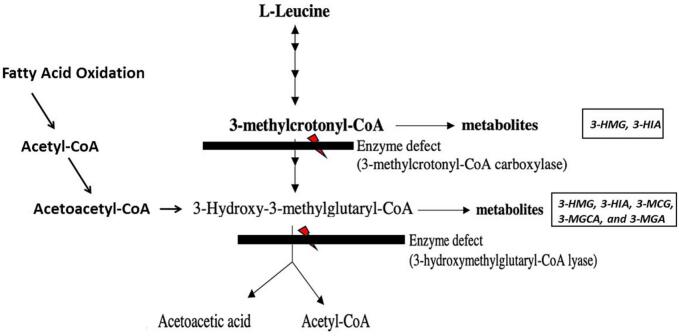
Overview of the involved metabolic pathways in the 3-methylcrotonyl-CoA carboxylase (3MCC 3-Hydroxy 3-Methylglutaryl-Coa Lyase (HMG-CoA Lyase) metabolism. *3-HMG: 3-hydroxy-3-methylglutaric acid, 3-HIA: 3-hydroxyisovaleric acid, 3-MCG: 3-methylcrotonylglycine, 3-MGCA: 3-mehylglutaconic acids, and3-MGA: 3-methylglutaric acids.*

In cases where C5OH is initially elevated, urine organic acid tests can confirm the diagnosis, and distinguish false positives. Specific urinary metabolite patterns can help identify the underlying disorder. For instance, 3-MCC deficiency is characterized by increased urinary excretion of 3-hydroxyisovalerate and 3-methylcrotonylglycine. HMG-CoA lyase deficiency patients show elevated levels of 3-hydroxy-3-methylglutaric acid, 3-hydroxyisovaleric acid, 3-methylcrotonylglycine, 3-mehylglutaconic acids, and 3-methylglutaric acids. β-ketothiolase deficiency is marked by elevated 2-methyl-3-hydroxybytyrate, 2-methylacetoacetate, and tiglyglycine (Fig. 1). Finally, MCD is diagnosed by elevated levels of methylcitric acid, 3-methylcrotonylglycine, tiglyglycine, lactic acid, 3-hydroxypropionic acid, and 3-hydroxyisovaleric acid [[10], [11], [12]].

3-Methylcrotonyl-CoA carboxylase (3-MCC) deficiency is relatively common compared to other screened organic acidurias in many countries, with an estimated incidence of approximately 1 in 50,000 [13]. In Saudi Arabia, 3-MCC ranks as the eighth most prevalent disease among 16 different conditions, with a prevalence of 1 in 16,847 [14]. Most cases of 3-MCC deficiency, including the majority of identified cases, do not typically require medical intervention, with various studies suggesting it is a benign condition [15]. Conversely, other disorders with elevated C5OH levels are rare, and symptoms in affected newborns often manifest shortly after birth [16]. Some non-specific symptoms observed in 3-MCC patients, such as intellectual disability and attention deficit disorder, may be influenced by genetic factors beyond the primary causative genes [17]. In light of these findings, reduce costs and alleviate parental concerns, some countries have adjusted their NBS programs. Some have opted not to report 3-MCC cases due to their benign nature, while others have removed C5OH as a screening marker altogether [18].

HMG-CoA lyase deficiency is characterized by recurrent metabolic crises, often presenting as non-ketotic hypoglycemia. These crises typically emerge in the neonatal period or later in infancy, resembling a severe metabolic state akin to Reye's syndrome [19]. The global prevalence of this condition is less than 1 in 100,000 live births, while in Saudi Arabia, the incidence is approximately 1 in 55,357, which is roughly double the global average [20].

This study aims to utilize the Inborn Errors of Metabolism Information System to gather data on the outcomes of high C5OH levels, ascertain the incidence of various diseases, and establish a correlation between specific diagnoses and outcomes with elevated C5OH levels in NBS.

## 2 Methods

### 2.1 Study design, setting, and sample size

We conducted a retrospective analysis of NBS-positive cases for C5OH-related diseases in the NBS information system of King Abdulaziz Medical City (KAMC) from May 2011 to December 2023. In the KAMC laboratory, the cut-off values for all parameters were determined using data from 74,000 patients analyzed in newborn screening program. Additionally, the laboratory engaged in the CDC's proficiency testing program to ensure ongoing monitoring of these established cut-off values. The C5OH level cutoff value was revised in 2020 to 0.50 μmol/L, as recommended by Huang et al., was used to define clinical disease ranges and multiple ratios for various analytes [21]. This approach provided excellent specificity and sensitivity for early detection with samples that were representative of the local population [22]. All cases underwent urine organic acid confirmation, and the diagnosis was confirmed by molecular analysis in all but three cases. In some cases, a molecular target test (Sanger) was used to confirm the diagnosis due to a close relative's positive NBS test, which was confirmed to be a C5OH-related disorder by the molecular test.

## 3 Results

### 3.1 Demographics

From May 2011 to December 2023, a total of 110,787 samples were received for NBS at KAMC in Riyadh, Saudi Arabia. The samples were analyzed through the Inborn Metabolic Disorders Information System (IBEM-IS), which identified all relevant cases. Out of these, 31 samples initially tested positive for C5OH, with 15 of them (48 %) ultimately confirmed as true positives. These true positive cases included ten males and five females, with 65 % of them having consanguinity and 30 % having a family history of the disorder. The age of the subjects at the time of inclusion in the database ranged from 3 to 13 years old, with an average age of 8 years. The follow-up visits after enrollment had an average duration ranging from 3 to 10 years.

### 3.2 Identification and case selection

In the concluding analysis, 15 cases were taken into account, and a quantitative measurement for the NBS C5OH level was obtained. The C5OH levels in the first NBS ranged from 0.84 to 11.7 (Cutoff value of 0.73 μmol/L), while the C5OH levels in the second NBS ranged from 0.87 to 15.1 (Cutoff value of 0.73 μmol/L). However, there was no significant difference in the NBS C5OH level among the various disorders. Among the 15 confirmed cases of C5OH-related disorders, 11 cases were identified as 3-MCC deficiency, and the remaining were identified as HMG-CoA lyase deficiency (Table 1).

**Table 1:** Patients clinical, biochemical, and molecular information.

Case	Current age (y) /Gender	1st NBS C5OH	2nd NBS C5OH	Final diagnosis	Neonatal symptoms	Metabolic crises	Growth	Diet	Medication	Gene/ Variant	Consanguinity	Family History	Outcome
		(Cut-off 0.73 μmol/L)											
1	13 y Male	11.7	15.1	3-MCC	Complex CHD	No	Normal	Regular	Carnitine at time of cardiac surgery	Homozygous/ *MCCC2*c.127C > T p.Gln43* Pathogenic	Yes	No	Normal
2	11 y Male	5.53	8.1	3-MCC	No	No	Normal	Regular	No	Homozygous/ *MCCC1* c.1808dupA p.N603Kfs*5 Variant of unknown significant	No	No	Normal
3	9 y Female	3.91	4.85	3-MCC	No	No	Normal	Regular	No	Homozygous*/ MCCC1*c.980C > G p.(Ser327*) Pathogenic	No	No	Normal
4	8 y Male	6.24	5.51	3-MCC	No	No	Normal	Regular	No	ND	Yes	No	Normal
5	8 y Female	1.00	0.99	3-MCC	No	No	Normal	Regular	No	Compound heterozygous/*MCCC2* c.1015G > A p. & c.281 + 5G > T p.(Val339Met) Pathogenic & likely pathogenic respectively	Yes	No	Normal
6	8 y Female	7.23	6.22	3-MCC	No	No	Normal	Regular	No	ND	Yes	No	Normal
7	7 y Male	7.57	9.11	3-MCC	No	No	Normal	Regular	No	Homozygous/ *MCCC2* c.1147 A > T p.(Lys383*) Pathogenic	Yes	Yes	Normal
8	7 y Male	0.84	0.87	3-MCC	No	No	Normal	Regular	No	ND	No	Yes	Normal
9	6 y Male	5.03	4.64	3-MCC	No	No	Normal	Regular	No	Homozygous/ *MCCC2* c.1151delG p.(Gly384Valf*54) Likely pathogenic	Yes	Yes	Normal
10	4 y Female	1.48	1.82	3-MCC	No	No	Normal	Regular	No	Homozygous/ *MCCC1* c.239G > A p.(Ser80Asn) Variant of unknown significant	No	No	Normal
11	3 y Male	4.43	6.16	3-MCC	No	No	Normal	Regular	No	Homozygous/ *MCCC1* c.1132C > T p.(Gln378*) Pathogenic	Yes	No	Normal
12	7 y Female	1.01	1.48	HMG-COA lyase	Metabolic acidosis, hyperammonemia, and hypoglycemia	Yes	Normal	Regular	Carnitine	Homozygous/ *HMGCL* c.122G > A (p.Arg41Gln) Pathogenic	Yes	Yes	Normal
13	4 y Male	1.65	2.24	HMG-COA lyase	Metabolic acidosis, hyperammonemia, and hypoglycemia	Yes	IUGR / FTT	Regular	Carnitine	Homozygous/ *HMGCL* c.122G > A (p.Arg41Gln) Pathogenic	Yes	Yes	Delayed
14	4 y Male	0.94	1.43	HMG-COA lyase	No	No	Normal	I-Valex-2	Carnitine	Homozygous/ *HMGCL* c.122G > A (p.Arg41Gln) Pathogenic	Yes	No	Normal
15	3 y Male	2.24	2.0	HMG-COA lyase	No	Yes (Mild)	Normal	Regular	Carnitine	Homozygous/ *HMGCL* c.122G > A (p.Arg41Gln) Pathogenic	No	No	Normal

3-MCC: 3-methylcrotonyl-CoA carboxylase, HMG-CoA lyase: 3-hydroxymethyl-3-methylglutaryl-coenzyme A lyase, NBS: Newborn Screening,

### 3.3 Confirmatory and molecular testing

In all detected cases, the diagnosis was conclusively established. Initially, a urine sample was obtained from every patient, which suggested the possible existence of C5OH-related disorders. However, an enzyme value was not determined for any of the patients. The subsequent step entailed a molecular examination where samples were dispatched for specific gene sequencing based on the urinary organic acid results, except for three cases with 3-MCC. Both genes *MCCC1* and *MCCC2* were impacted equally (4 cases each) with distinct mutation spectra, all of which were homozygous except for one case (case #5) which was compound heterozygous. The founder mutation (*HMGCL* c.122G > A (p.Arg41Gln)) was identified in all cases of HMG-CoA lyase deficiency (Table 1).

### 3.4 Neonatal period

None of the patients with 3-MCC deficiency showed any neonatal symptoms or complications, and there was no need for postnatal admissions. The first patient, a male infant with a complex congenital heart condition consisting of a single ventricle, atrial sinus inversus, mitral and pulmonary valve atresia, left ventricular atrophy, transposition of the great artery, and ventricular septal defect, did not exhibit any signs of metabolic decompensation. However, another patient with HMG-CoA lyase deficiency (patient #12) experienced apnea soon after birth, necessitating intubation and treatment for metabolic decompensation, which included metabolic acidosis, hyperammonemia, and lactic acidosis.

### 3.5 Clinical outcome

Patients with 3-MCC deficiency, including Case No.5 who followed a protein-restricted diet for the first three years of life, showed normal growth and development, with no neurological delays or recurrent diseases. No patients with 3-MCC deficiency were currently using carnitine supplements, except for one case who took carnitine before cardiac surgery at a young age.

In the HMG-CoA lyase deficiency group, one patient (case #12) had short stature, and two-thirds of the patients had experienced recurrent hypoglycemia. However, the overall prognosis was good for most patients, except for one case (case #12) who had global developmental delay due to recurrent metabolic crisis (hypoglycemia and severe metabolic acidosis). Family history of seizures, ASD, and ADHD, indicating a possible coexisting disease, however extensive genetic work-up did not revealed any additional diagnosis. Another patient in this group (case #14) was on a protein-restricted diet in the first two year of life, and all patients were taking carnitine supplements, although there was insufficient data on carnitine levels before and after its use. A five-year-old girl in the group also had delayed speech.

## 4 Discussion

While a group of conditions may be flagged by an NBS indicator, it is important to note that these conditions can vary significantly in terms of severity and symptoms. To gain a better understanding of how to follow up with newborns who have elevated C5OH levels, a study was conducted on infants and families screened between January 2011 and December 2022. The study excluded cases diagnosed based on symptoms. The spectrum of C5OH-related disorders ranged from individuals showing no symptoms to those experiencing severe, life-threatening acidosis episodes, necessitating accurate evaluation of cases with abnormal C5OH levels. Currently, there are at least three commercial kits for the analysis of acylcarnitine in DBS, each with different C5OH recovery rates [21]. Establishing precise cutoff values for C5OH is challenging due to its complexity and involvement in various metabolic pathways. When determining cutoff values, it is crucial to consider population data averages and maintain clear communication between screening labs and medical teams to minimize false positives without missing potential cases. [9,22]. The C5OH/C8 ratio is used as a primary marker for certain C5OH-positive cases. Additionally, quantifying 3-hydroxybutyrylcarnitine (C4OH) is important in ß-ketothiolase deficiency due to similarities in compound structures and ionization efficiency [23,24]. New potential diagnostic biomarkers for 3-hydroxy-3-methylglutaryl-CoA lyase deficiency have been identified through untargeted metabolomics. [25].

In this study, all 3-MCC cases remained asymptomatic, with ages ranging from 2 to 12 years. There was no observed correlation between C5OH levels at the NBS and in the clinical outcomes, nor was there a clear genotype-phenotype correlation. All individuals with HMG-CoA lyase deficiency displayed distinct variations in their clinical presentation. Some patients experienced metabolic decompensation due to illness, which required hospitalization and could result in long-term neurological complications. The four cases in this study were all homozygous for the mutation HMGCL c.122G > A (p.Arg41Gln). All patients underwent regular follow-up, however two cases (Case #12 and Case #13) exhibited some symptoms that necessitated regular and closer monitoring, while the other two cases did not show any neurological symptoms like developmental delay, speech delay, or seizures. However, the genotype-phenotype correlation was not apparent. Non-ketotic hypoglycemia was observed in symptomatic cases, while C5OH levels varied and did not correspond to symptomatic or asymptomatic cases. This indicates that HMG-CoA lyase deficiency is a diverse condition with unpredictable outcomes, so early detection of the disease could lead to appropriate metabolic management, which may contribute to a favorable cognitive outcome. [20].

In conclusion, our findings offer a new insight into NBS, using C5OH as a marker for certain disorders. Considering recent findings from this study and many NBS programs, modifying the C5OH marker to not to report 3-MCC cases particularly to reduce costs and alleviate parental concerns is recommneded. However, due to the rarity of these diseases, further prospective large-scale studies are needed to establish the feasibility of this approach and to create an optimal screening algorithm for C5OH-related conditions. Additionally, the development of a standardized methodology for second-tier tests could enhance diagnostic precision and enable faster diagnosis in NBS.

## Ethics approval

This study was approved by the 10.13039/501100013302King Abdullah International Medical Research Center. IRB (#RC20/621/R).

## Consent for publication

Written informed consent to participate and consent for publication were obtained from the study participants' parents or guardians before study commencement for the patients included in the table-1.

## Funding

This study received no specific funding from any financial support agency either public or commercial and not-for-profit sectors.

## CRediT authorship contribution statement

**Fuad Al Mutairi:** Writing – review & editing, Writing – original draft, Visualization, Validation, Supervision, Resources, Project administration, Methodology, Formal analysis, Data curation. **Randa Alkhalaf:** Writing – original draft, Validation, Data curation. **Abdul Rafiq Khan:** Validation, Methodology, Investigation, Formal analysis, Data curation. **Ali Al Othaim:** Validation, Methodology, Formal analysis, Data curation. **Majid Alfadhel:** Validation, Formal analysis.

## Declaration of competing interest

The authors declare the following financial interests/personal relationships which may be considered as potential competing interests:

Fuad AL Mutairi reports administrative support, equipment, drugs, or supplies, and writing assistance were provided by King Abdullah International Medical Research Center. If there are other authors, they declare that they have no known competing financial interests or personal relationships that could have appeared to influence the work reported in this paper.

## Data Availability

The authors confirm that the data supporting the findings of this study are available within the article.
